# Validation of carbon dioxide production (VCO_2_) as a tool to calculate resting energy expenditure (REE) in mechanically ventilated critically ill patients: a retrospective observational study

**DOI:** 10.1186/s13054-018-2108-8

**Published:** 2018-08-03

**Authors:** I. Kagan, O. Zusman, I. Bendavid, M. Theilla, J. Cohen, P. Singer

**Affiliations:** 1 Department of General Intensive Care and Institute for Nutrition Research, Rabin Medical Center, Beilinson Hospital Affiliated to Sackler School of Medicine, Tel Aviv University, Jabotinsky St 39, 49100 Petah-Tikva, Israel; 2 Department of Cardiology, Rabin Medical Center, Beilinson Hospital, Affiliated to Sackler School of Medicine, Tel Aviv University, Jabotinsky St 39, 49100 Petah Tikva, Israel; 3 Stanley Steyer School of Health Professions, Sackler School of Medicine, School of Nursing, Tel Aviv University, Haim Levanon Street 55, Ramat Aviv 6997801 Tel Aviv, Israel

**Keywords:** Indirect calorimetry, Oxygen consumption (VO_2_), Resting energy expenditure (REE), Carbon dioxide production (VCO_2_), Abbreviations, IC Indirect calorimetry, REE Resting energy expenditure, RQ Respiratory quotient, VCO_2_ Carbon dioxide production, VO_2_ Oxygen consumption

## Abstract

**Background:**

Indirect calorimetry (IC) measurement is considered the gold standard for the assessment of resting energy expenditure (REE). It is based on the measurement of oxygen and carbon dioxide consumption (VO_2_ and VCO_2_, respectively). However, its use is limited by cost and technical issues. It has been proposed that, in critically ill patients, the analysis of VCO_2_ obtained from the ventilator alone may be used as an accurate method to assess REE in ventilated patients. This retrospective study aimed to assess the accuracy of VCO_2_ measurement alone in the determination of REE.

**Methods:**

This was a retrospective study conducted at the general intensive care unit of a single university-affiliated tertiary medical center. Patients included were invasively ventilated and their REE was measured by using IC. The respiratory quotients (RQs) were set at 0.8, 0.85, and 0.89. Data were collected from computerized patient files. REE obtained from the ventilator by using VCO_2_ (REE-VCO_2_) alone was compared with REE obtained from IC (REE-IC).

**Results:**

Measurements were obtained for 80 patients, and 497 REE-IC measurements were compared with REE-VCO_2_ obtained at the same time. The mean REE-IC was 2059.5 ± 491.7 kcal/d. The mean REE-RQs corresponding to RQs of 0.80, 0.85, and 0.89 were 1936.8 ± 680.0, 2017.8 ± 708.8, and 2122.1 ± 745.4 kcal/d, respectively. REE-VCO_2_ derived from an RQ of 0.85 had the lowest mean difference from REE-IC. Whereas accuracy was higher using an RQ of 0.85, agreement (between 85% and 115%) was highest using an RQ of 0.89.

**Conclusions:**

The level of agreement of REE obtained from VCO_2_ readings with REE obtained from IC was generally low. IC continues to be the recommended method for REE assessment.

## Background

The use of indirect calorimetry (IC) to assess resting energy expenditure (REE) based on the measurement of oxygen consumption (VO_2_) and carbon dioxide production (VCO_2_) has been demonstrated in several studies and meta-analyses to be more accurate when compared with predictive equations [1–4]. Since its use remains limited (mainly for financial and technical reasons), the use of VCO_2_ alone has recently been suggested as an alternative method to calculate REE in children as well as in adults in various settings [5–7]. This method is based on the Weir equation, where VO_2_ is not measured but is derived as being equal to VCO_2_/RQ, where RQ is the respiratory quotient which in turn is derived from VCO_2_/VO_2_ [8]. This is defined as the REE-VCO_2_. However, the level of accuracy of REE-VCO_2_ is uncertain, and a comparison between REE derived from the VCO_2_ calculated from the calorimeter and REE derived from VO_2_ and VCO_2_ from the mechanical ventilator has shown significant differences [8]. The RQ value may vary according to substrate utilization and therefore the REE calculation may differ accordingly. The present study was conducted to compare the REE-VCO_2_ compared with the REE derived from IC (REE-IC).

## Methods

This retrospective observational study was performed at a 16-bed mixed medical-surgical intensive care department of a university-affiliated tertiary hospital. Data were derived from the database of a computerized information system (MetaVision ICU^®^, *i*MDs*oft*, Tel Aviv, Israel). All patients from 2003 to 2015 who underwent IC measurements (Deltatrac II, Datex-Ohmeda, part of GE Healthcare, Madison, WI, USA) were included in this study. The calorimeter was calibrated with ethanol on a monthly basis and for test gases (ambient air and O_2_ 95% and CO_2_ 5%) before all measurements. Prior to testing, patients were required to be in stable condition for at least 30 min, ventilated with fraction of inspired oxygen (FiO_2_) of less than 60%, and positive end-expiratory pressure (PEEP) of less than 10 cm H_2_O without any discernable air leaks. Only stable measurements for at least 20 min were considered acceptable. Oxygen consumption and CO_2_ production were measured, and the RQ and REE were calculated by using the Weir equation. The timing and number of IC measurement per patient were determined by the treating physician. All patients hospitalized between 2014 and 2017 who had an IC measurement and a simultaneous VCO_2_ measurement obtained from the ventilator (Evita 4, Dräger, Lübeck, Germany) were included in the study. Demographic data noted included age, sex, weight, height, body mass index, and Sequential Organ Failure Assessment (SOFA) score. VO_2_, VCO_2_, REE, and RQ were obtained from the IC measurements [9]. In addition, VCO_2_ was obtained from the ventilator over 6-h blocks and was used to calculate REE-VCO_2_. RQ values were chosen arbitrarily as 0.8, 0.85, and 0.89 because these values are the most commonly used values to derive REE from VO_2_ [10].

The study was approved by the institution’s review board, and informed consent was waived since the data were recorded retrospectively.

REE-IC and REE-VCO_2_ were compared by using mean difference, standard deviation, percentage of error, percentage of difference, correlation, concordance, agreement with the equation 5.5 × VCO_2_ × 1.44 (within 85% and 115% of measured REE), and tight agreement within 95% and 105% of measured REE). In addition, we used ridge regression, a form of penalized linear regression, to estimate REE from VCO_2_ measurements derived from the ventilator. A Bland–Altman graph and a scatter plot were performed to study the precision of the method.

## Results

Eighty patients were included in the study, and 497 REE-IC measurements with a corresponding mean of the 6-h block VCO_2_ were performed. The median number of measurements per patients was 3 (interquartile range of 2–7). Patients’ characteristics are presented in Table 1.

**Table 1:** Patient characteristics

*N* = 80	Mean	Standard deviation
Sex	53% male, 47% female	–
Age, years	55.6	22.5
SOFA score	6.6	3.5
APACHE II score	22.5	7.0
Weight, kg	82.4	21.7
Height, m	1.70	0.10
Body mass index, kg/m^2^	28.7	8.7
Enteral energy delivered, kcal/d	1278	654
Parenteral energy delivered, kcal/d	333	439

*Abbreviations*: *APACHE* Acute Physiology and Chronic Health Evaluation; *SOFA* Sequential Organ Failure Assessment

The mean caloric intake from enteral sources was 1278 ± 654 kcal, whereas the mean caloric intake from parenteral sources was 333 ± 439 kcal. Mean protein intake was 68 g/day and comprised 18% of calories, and 38% of energy was derived from lipids and 44% from carbohydrates.

The mean REE-IC was 2059.5 ± 491.7 kcal/d, and the mean RQ was 0.75 ± 0.07. The mean 6-h block VCO_2_ was 244.5 ± 85.9 mL/min. The mean REE-RQs corresponding to RQs of 0.80, 0.85, and 0.89 were 1936.8 ± 680.0, 2017.8 ± 708.8, and 2122.1 ± 745.4 kcal/d, respectively.

**Fig. 1:**
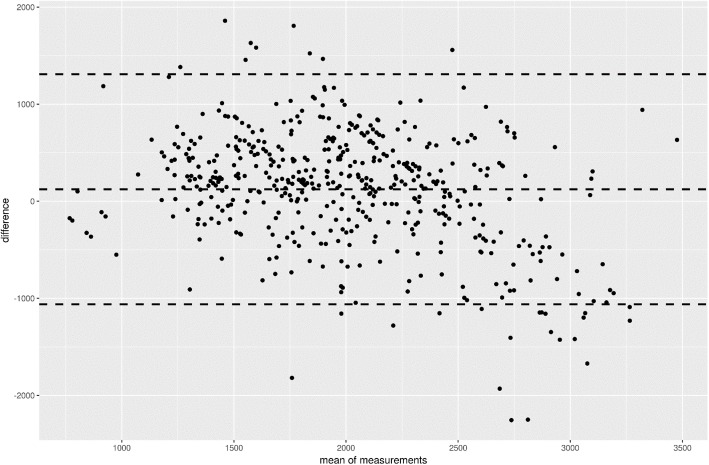
Bland–Altman graph representing resting energy expenditure derived from VCO_2_ (REE- VCO_2_) in comparison with REE obtained from indirect calorimetry (REE-IC)

**Fig. 2:**
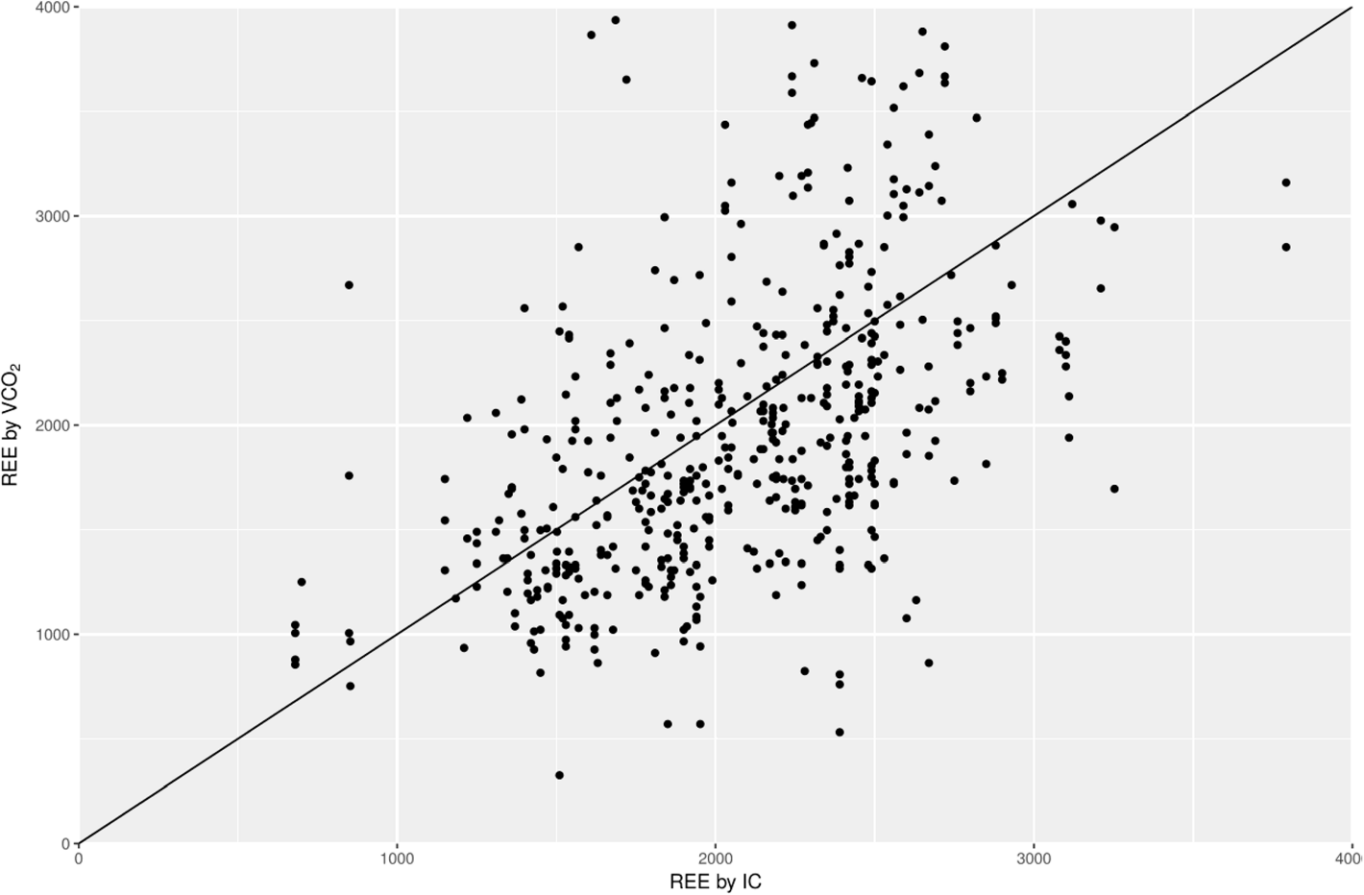
Correlation between resting energy expenditure derived from VCO_2_ (REE-VCO_2_) and REE calculated from indirect calorimetry measurements (REE-IC) presented as a scatter plot

The VCO_2_ performance is presented in Table 2. The mean RQ of all patients in the intensive care unit who had REE-IC measurements (*n* = 3326) was 0.79 ± 0.11 (personal data). A Bland–Altman figure (Fig. 1) shows a wide variability but without a consistent bias suggesting that the measurement could widely under- or overestimate REE. A scatter plot (Fig. 2) confirms these findings.

**Table 2:** The VCO_2_ performance

	Mean difference	Standard deviation	% Error	% Difference	Correlation	Concordance	Agreement^a^	Tight agreement^b^
VCO_2_ with 0.89 RQ^c^	− 122.78	604.92	0.24	0.16	0.51 (0.44–0.57)	0.37 (0.27–0.47)	0.37	0.11
VCO_2_ with 0.85 RQ	−41.77	625.57	0.24	0.16	0.51 (0.44–0.57)	0.37 (0.27–0.47)	0.4	0.13
VCO_2_ with 0.80 RQ	62.54	652.95	0.25	0.16	0.51 (0.44–0.57)	0.37 (0.26–0.47)	0.42	0.16
VCO_2_ with 0.75 RQ	178.17	684.37	0.26	0.16	0.51(0.44–0.57)	0.35(0.25–0.45)	0.43	0.18

^a^Within 85% and 115% of measured REE

^b^Within 95% and 105% of measured REE

^c^Equivalent to REE calculation of 5.5*VCO_2_*1.44

*Abbreviations*: *REE* resting energy expenditure, *RQ* respiratory quotient, *VCO*_*2*_ carbon dioxide production

REE-VCO_2_ derived from an RQ of 0.85 had the lowest mean difference from REE-IC and the same percentage error, difference, correlation, and concordance with REE-VCO_2_-RQ derived from RQs of 0.80 and 0.89. The agreement (between 85% and 115%) was slightly lower than for REE-RQ derived from 0.89 but better than REE-RQ derived from 0.80 with the same results as the tight agreement.

When we estimated REE using only VCO_2_ measurements made by the calorimeter using penalized regression as described, VCO_2_ was found to be significantly associated with REE-IC (*P* <0.001, R^2^ = 0.84). A simplified formula (i.e. REE = 135 +8*VCO_2_) was then derived. Using this formula resulted in a difference of 52 kcal, standard deviation of 220 kcal, 9% percent error, 6% percent difference, 92% correlation, 72% concordance, 82% agreement, and 33% tight agreement. However, application of this formula to the 80 ventilated patients resulted in similar performance to predefined RQ (agreement of 0.43).

## Discussion

Our study shows a small mean difference in the REE derived from the REE-VCO_2_ using the derived equation but with low agreement when compared with the gold-standard REE obtained by IC. In addition, we demonstrated the ability of VCO_2_ to predict REE based on measurements by the calorimeter and specific regression with very high accuracy (agreement). This represents the “ceiling” of possibility of using VCO_2_, but it remains unclear whether this applies to VCO_2_ measurements made by the ventilator. Using VCO_2_ to estimate energy expenditure is challenged by a medical hazard: the quite unpredicted error as compared with the gold standard. If mean (median) values presented may perhaps be acceptable, the larger scatter is not. Individual subjects may not have an inaccurate evaluation, as clearly shown in Figs. 1 and 2).

Stapel et al. [5] found that 10% and less than 15% accuracy rates of REE-VCO_2_ were 61% and 79%, respectively. Less than 25% and less than 30% inaccuracy rates of REE-VCO_2_ were 2% and 0%, respectively. There results were superior to those derived from predictive equations. The differences between their results and those we have shown may be explained by methodological details. First, the VCO_2_ measurements in the study by Stapel et al. were obtained as a mean of 24 h and not from a block of 6 h as in our study. In our study, VCO_2_ measurements were obtained as a mean of 6 h and compared with a measurement of 20 min using the Deltatrac II instrument. Second, we used a different ventilator (Dräger) from that used in the study by Stapel et al. (Maquet, Rastatt, Germany), and since our study was retrospective, there was no calibration before each measurement. Finally, we did not use an RQ according to nutritional intake but rather three values representing common clinical states, including the RQ of 0.86 used in the study by Stapel et al., which is the most frequent RQ resulting from nutrition. The use of RQ according to nutrition intake has been applied by others in children [6]. Mehta et al. used an RQ defined by macronutrient administration defined as VCO_2_RQmacro (kcal/day) = [3.941(VCO_2_/RQmacro) + 1.106(VCO_2_)] × 1440. VCO_2_-REE was obtained using an RQ of 0.9 using the equation: REE = [3.941 (VCO_2_/0.89) + 1.106(VCO_2_)] × 1440 = [4.428(VCO_2_) + 1.106(VCO_2_)] × 1440 = 5.534(VCO_2_) × 1440. The authors described a mean bias (limits) for agreement between measured REE and REE-VCO_2_ or VCO_2_-RQmacro of −0.6 (−14.4 to 13.1) and −2.0 (−42.9 to 38.8), respectively, using REE-VCO_2_ in comparison with REE-IC. When patients were classified as hypometabolic or hypermetabolic according to REE-IC divided by the Schoeffield equation, the accuracy, sensitivity, and specificity were 0.86, 1.00, and 0.83 and 0.82, 0.62, and 1.00, respectively. The conclusions of the authors were in favor of using an RQ of 0.89 (the mean of the measurements in their study) and evaluating REE from VCO_2_ alone as being an acceptable method. Others [7] compared REE-IC obtained by a GE module giving continuous VO_2_, VCO_2_, and REE to an REE-VCO_2_ obtained by VCO_2_ and a fixed RQ of 0.85. In most patients (89%), accuracy (± 10%) was obtained. However, the authors noted the problems linked to variations in minute volume limiting the validity of the measurements. These variations have been described previously [11], and a stable ventilation setting has been a condition to validate REE measurements. Finally, Oshima et al. [8] used another methodology to compare REE-IC with REE-VCO_2_. All measurements (VO_2_, VCO_2_, and REE) were obtained from IC measurements and not from the ventilator. They compared REE-IC with REE-VCO_2_ obtained using an RQ of 0.85 or RQ related to nutritional intake. Mean biases (lower, upper 95% confidence intervals) for REE-VCO_2__0.85 and REE-VCO_2__FQ (derived from Food Quotient) were −21 kcal/d (−41, 1) and −48 kcal/d (−67, −28), respectively. The limits of agreement in Bland–Altman plots were (+314, −356) and (+272, −367), respectively. The 5% accuracy rates compared with REE-IC were 46.0% and 46.4%, and 10% accuracy rates were 77.7% and 77.3%, respectively. The authors confirmed the finding from McClave et al. [12] that RQ is neither a reliable indicator of the feeding status nor strongly associated with non-nutritional factors such as mode of ventilation and acid-base disturbances. RQ based on VCO_2_ cannot be as accurate for REE evaluation when compared with VO_2_-based equations. The authors concluded that REE-VCO_2_ was not accurate enough to be considered an alternative to IC. Using the same methodology, Mouzaki et al. [12] also evaluated REE-VCO_2_ compared with REE-IC in a cardiac pediatric population and reached results similar to those of Oshima et al. Wide limits of agreement and a high percentage error suggested that the REE derived from VCO_2_ was inaccurate only when compared with the gold standard. These findings were explained by a large RQ distribution.

Our study has several limitations. It is retrospective and did not take into account sedation, ventilator types, and settings but used a VCO_2_ measurement over the 6 h preceding the assessment. The RQ examined used predefined common values and was not obtained according to nutritional intake. Although the sample size was comparable to that of other studies, it was small and single-centered. Finally, there is a limitation in the reliability of some calorimeters when compared with Deltatrac II. Sundström et al. [13] found higher limits of agreement when comparing Deltatrac II with the Quark device than Stapel et al. found between Datex and the VCO_2_ respirator-derived approach. Graf et al. [14] confirmed these increased limits of agreement when comparing Quark and the CCM device with Deltatrac II. The reliability of the reference calorimetry therefore should be ensured.

## Conclusions

In this retrospective study, we did not find tight agreement between REE-IC and REE-VCO_2_ using various RQ values. Thus, for defining the appropriate calorie target in critically ill patients, IC remains the best tool. In recent years, great efforts have been made to develop easier-to-use and more accurate IC devices at lower costs, reflecting the clinicians’ needs. Some of them stand alone and some are integrated in monitors or ventilators. All of these new devices require careful evaluation and validation but will allow a more accurate evaluation of the energy expenditure of critically ill patients. Where IC is not possible, REE derived from VCO_2_ obtained from the ventilator may be the best alternative. However, it must be stressed that using VCO_2_ from the ventilator and an arbitrary RQ to derive REE remains less accurate than IC.
